# Intracellular Context Affects Levels of a Chemically Dependent Destabilizing Domain

**DOI:** 10.1371/journal.pone.0043297

**Published:** 2012-09-12

**Authors:** Mark A. Sellmyer, Ling-chun Chen, Emily L. Egeler, Rishi Rakhit, Thomas J. Wandless

**Affiliations:** Department of Chemical and Systems Biology, Stanford University, Stanford, California, United States of America; Consejo Superior de Investigaciones Cientificas, Spain

## Abstract

The ability to regulate protein levels in live cells is crucial to understanding protein function. In the interest of advancing the tool set for protein perturbation, we developed a protein destabilizing domain (DD) that can confer its instability to a fused protein of interest. This destabilization and consequent degradation can be rescued in a reversible and dose-dependent manner with the addition of a small molecule that is specific for the DD, Shield-1. Proteins encounter different local protein quality control (QC) machinery when targeted to cellular compartments such as the mitochondrial matrix or endoplasmic reticulum (ER). These varied environments could have profound effects on the levels and regulation of the cytoplasmically derived DD. Here we show that DD fusions in the cytoplasm or nucleus can be efficiently degraded in mammalian cells; however, targeting fusions to the mitochondrial matrix or ER lumen leads to accumulation even in the absence of Shield-1. Additionally, we characterize the behavior of the DD with perturbants that modulate protein production, degradation, and local protein QC machinery. Chemical induction of the unfolded protein response in the ER results in decreased levels of an ER-targeted DD indicating the sensitivity of the DD to the degradation environment. These data reinforce that DD is an effective tool for protein perturbation, show that the local QC machinery affects levels of the DD, and suggest that the DD may be a useful probe for monitoring protein quality control machinery.

## Introduction

Proteins are important for almost every cellular process. Accordingly, a significant portion of modern biology is devoted to studying the production and interactions of proteins. As biologists gain a quantitative understanding of the timing, concentration, and spatial localization important for protein function, molecular tools allowing for precise cellular perturbations are vital [1]. Consequently, we developed a small, inherently unstable protein domain based on the FK506- and rapamycin-binding protein (FKBP12), termed a destabilizing domain (DD) [2]. This instability can be conferred to a genetically fused protein of interest, and the resulting fusion protein is rapidly degraded in the absence of stabilizing ligand. The addition of a specific small molecule ligand, Shield-1, can rescue the fusion protein from degradation in a rapid, dose-dependent, and reversible manner. This system has been widely applied in variety of cell types and organisms [3], [4], [5], [6], [7], [8], [9], [10], [11].

The definitive mechanism of DD regulation has not been fully elucidated, although it is known that cytoplasmic DD degradation is mediated by the ubiquitin-proteasome system [12]. By targeting DD fusions to the endoplasmic reticulum (ER) we found Shield-1 could regulate extracellular, secreted proteins over 1–2 orders of magnitude [3]. However, we also noticed elevated levels of DD fusions that co-localized with ER in the absence of Shield-1. These observations precipitated the idea that the local degradation and quality control machinery specific to each subcellular locale may significantly affect DD levels and ligand-dependent regulation, thus warranting further investigation of the technology.

In the last 30 years considerable progress has been made toward determining the machinery of protein homeostasis in the cell. Most notably the ubiquitin-proteasome system (UPS) is a general mechanism for protein degradation in the cytosol and degrades most cytoplasmic substrates [13], [14]. The UPS functions via a series of protein interactions that modify substrates with ubiquitin and targets them to the proteasome for degradation. Recently the focus has increased on compartmental degradation such as ER-associated degradation (ERAD). This work has led to the discovery of two important sets of proteins that are integral to ER compartment homeostasis and which function in concert with ER chaperones and folding enzymes, such as BiP, calnexin, calreticulin, and EDEM. The first set is uniquely devoted to ERAD and the biochemical interactions that remove misfolded substrates from the ER [15]. The second set of proteins controls the ER unfolded protein response (UPR) and allows the cell to adapt to misfolded substrates in the ER [16]. Similarly, the mitochondria has its own molecular chaperones, proteases, and mechanisms of dynamic response to misfolded protein stress [17]. As the degradation of the DD appears to be proteasome dependent, and the UPS functions within the cytoplasm, we sought to test the behavior of the DD in various cellular compartments in conjunction with perturbants that modulate protein production, degradation, and local protein QC machinery.

Our results reinforce our previous work that the DD effectively regulates protein levels in the cytoplasm, nucleus, and through the ER. We show for the first time that the ER and mitochondria have limited ability to recognize and/or degrade the DD based on fluorescence microscopy, flow cytometry and immunoblot in the absence of Shield-1. The induction of protein quality control machinery in the ER significantly reduces the basal levels of the DD protein in the ER in the absence of Shield-1 suggesting that the ER, unlike the cytoplasm, is tolerant of elevated levels of DD. To further explore whether the DD could initiate the ER UPR upon Shield-1 washout (*i.e.* switching from secretion to degradation of the DD), we show that the DD proteins in the ER were not capable of inducing the UPR as measured by XBP1 splicing. These studies provide insights into how efficiently the DD functions as a tool for protein perturbation in diverse cellular environments and can be affected by changes in the local degradation machinery.

## Results

We made several genetic constructs encoding fluorescent and luminescent proteins fused to the DD to test how each cellular compartment would respond to the DD and perturbation with Shield-1 (Table 1). We genetically fused the DD to the N-terminus of YFP to create the cytoplasmic, cDD, cell line. To generate a nuclear localized DD, nDD, we added the nuclear localization sequence from the SV40 large T-antigen to the N-terminus of the DD-YFP construct. In the mitochondria we tested both the N and C-terminal orientation of a DD relative to Venus fluorescent protein, mDDn and mDDc. The mitochondrial DD reporter constructs contain the mitochondrial matrix targeting sequence from aldehyde dehydrogenase 2 (ALDH2, [18]). The ER DD fluorescent reporter, eDD, was made by fusing the secretion signal from *Gaussia principis* secreted luciferase (GLuc, [19]) to the N-terminus of DD-GFP. To create an optical, secreted extracellular reporter protein, eDDs, a functional GLuc was cloned in the place of the ER targeted GFP. Targeted DD constructs were introduced into HEK293 cell by retroviral transduction, and drug selection produced stable populations containing the DD (Figure S1).

**Table 1:** Targeted DD Reporters.

Compartment	Construct	Cell Line Name
Cytoplasm	DD-YFP	cDD
Nucleus	NLS-DD-YFP	nDD
ER (intracellular reporter)	LS-DD-GFP	eDD
ER (extracellular reporter)	LS-DD-GLuc	eDDs
Mitochondria (N-terminal DD)	MTS-DD-Venus	mDDn
Mitochondria (C-terminal DD)	MTS-Venus-DD	mDDc

The chemical dependence of the destabilizing domain allows the quantitative comparison of protein levels in each cell line after treatment with Shield-1 or vehicle control, and small molecule perturbants of the translation, degradation, secretion, and local quality control machinery. Only qualitative comparisons may be made between the raw fluorescence intensity values across the cell lines since targeting sequences affect the expression levels of the fusions, there are differences in retroviral transduction, and we used different variants of a fluorescent reporter protein. For example, we used Venus fluorescent protein for mitochondrial targeted mDDn and mDDc as it is more tolerant of acidic environments [20]. An additional caveat of these experiments is that we cannot be certain of the relative penetrance of Shield-1 into each compartment. Despite these limitations, our results can be generalized and used as a benchmark for future studies using the DD technology in subcellular compartments and suggest the importance of the local QC machinery for functional regulation.

### Cytoplasmic and nuclear destabilizing domains

**Figure 1:**
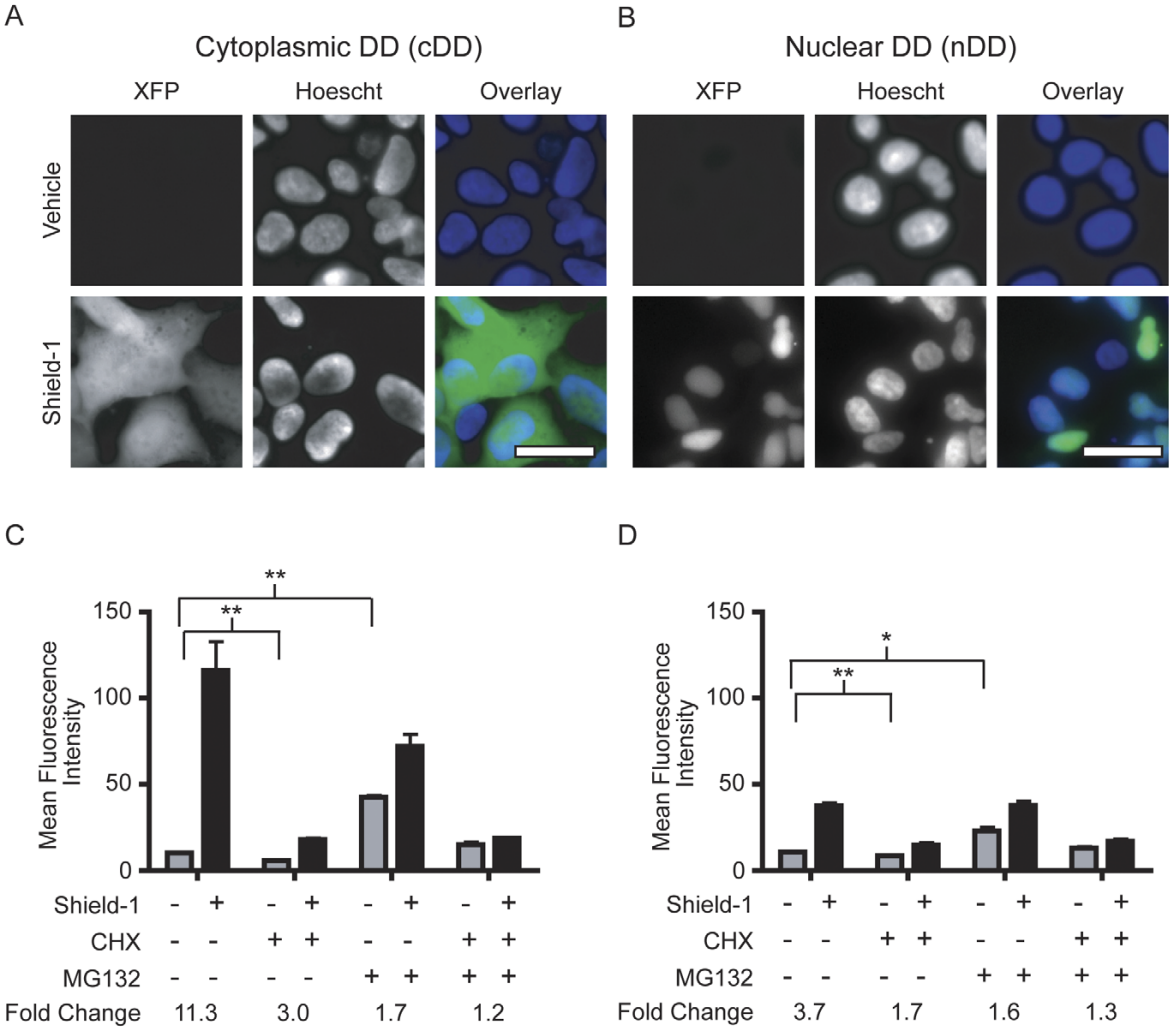
Destabilizing domains in the cytoplasm and nucleus. (A) Live cell fluorescence micrographs showing cDD cells after overnight treatment with vehicle control or Shield-1 (1 µM). The overlay shows DD-XFP (green) and Hoechst staining (blue) indicating the nuclear compartment. (B) Fluorescence micrographs of nDD cells treated as in (A). (C) Flow cytometry of cDD cells with Shield-1 (2 µM) or vehicle control after a 6 hour incubation. Cells were co-treated with cycloheximide (CHX, 5 µg/mL), MG132 (5 µM), or both CHX and MG132. (D) Flow cytometry of nDD cells with the same concentration of small molecule inhibitors as in (C). Micrograph scale bars indicate 10 microns. Error bars represent ± S.E.M. (n = 3). *p<0.05, **p<0.005.

We first tested the change in fluorescence in the cytoplasm and nucleus using cDD and nDD cells after treatment with vehicle or Shield-1 (1 µM). Both cDD and nDD cell lines displayed Shield-1 dependent fluorescence after overnight treatment using fluorescence microscopy (Figure 1A and B). cDD cells show diffuse cytoplasmic fluorescence while the nDD fluorescence colocalizes with Hoechst nuclear stain indicating localization of the fusion protein to the nucleus. Quantitative fluorescence levels were assayed using flow cytometry in cDD and nDD cells that were treated with vehicle or Shield-1 (2 µM) for 6 hours, a shorter time course allowing the concurrent treatment with other small molecule perturbants. DD fusions in the cytoplasm had a 11.3-fold induction of signal while fusions in the nucleus had a 3.7-fold induction (Figure 1C and D).

Chemical inhibitors of protein translation, cycloheximide (CHX), and the proteasome, MG132, were used to assess production and degradation of the DD fusions in the cytoplasm and nucleus. Each sample was treated with vehicle or Shield-1 and simultaneously treated with CHX (5 µg/mL), MG132 (5 µM), or co-treated with both for 6 hours. Fluorescence levels were quantified using flow cytometry (Figure 1C and D). CHX decreased the background fluorescence levels without Shield-1 treatment in cDD cells (p<0.005). MG132 increased background fluorescence levels (p<0.005), indicating decreased DD fusion degradation after proteasome blockade and supporting our previous data suggesting that destabilized fluorescent proteins are degraded via the UPS [2], [12]. In the presence of Shield-1 and MG132, fluorescence levels were lower than in cells treated with Shield-1 alone.

When cDD and nDD cells are treated with CHX, Shield-1 did not cause as drastic an induction of fluorescence, 3.0-fold and 1.7-fold respectively (Figure 1C and D). As expected, treating with both CHX and MG132 led to little Shield-1 dependent regulation of fluorescence. Neither untransduced cells nor cells constitutively expressing DD-free fluorescent protein showed significant changes in fluorescence when treated with any of the small molecules as described above (Figure S2).

### Mitochondrial destabilizing domains

**Figure 2:**
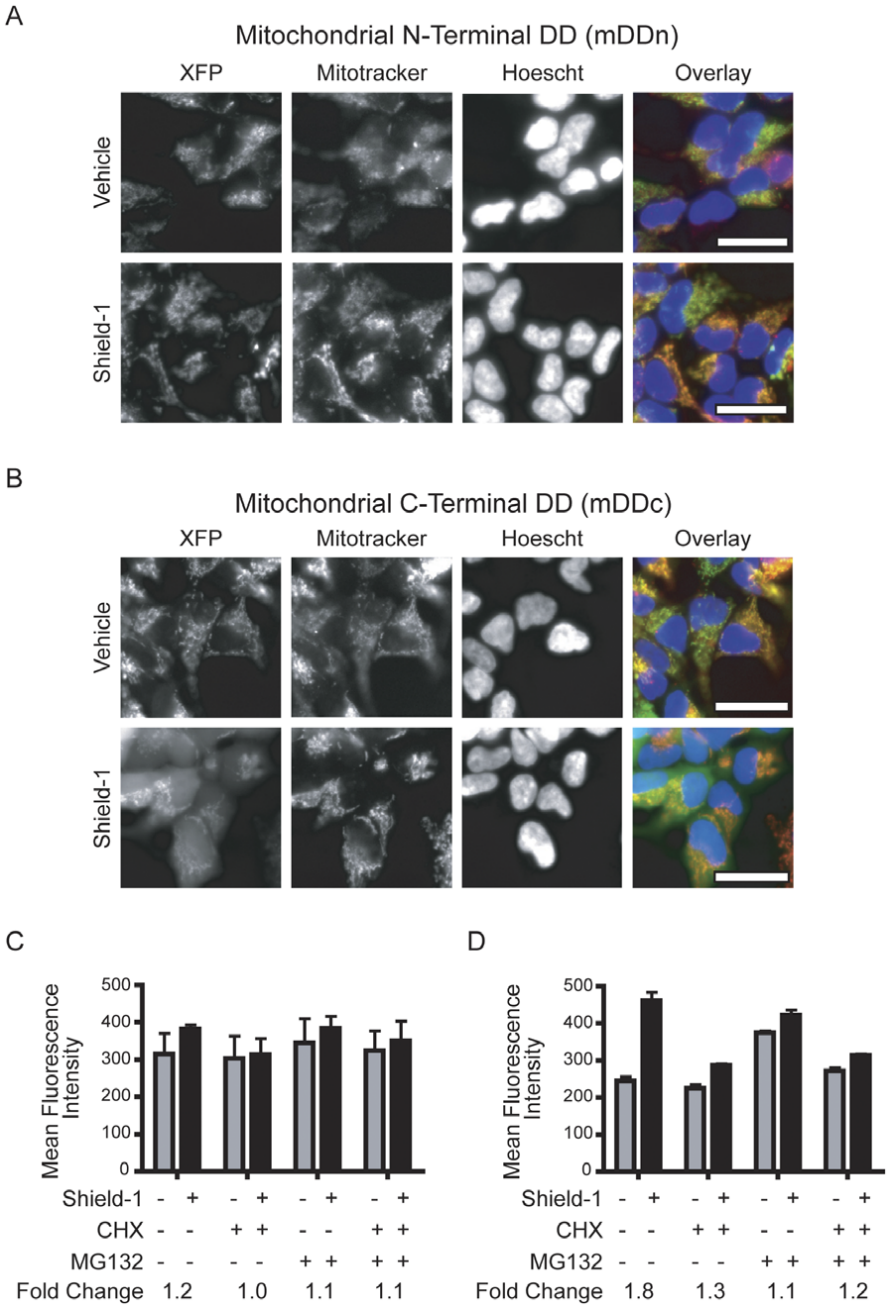
N- and C-terminal DDs targeted to the mitochondria. (A) Fluorescence micrographs of mDDn cells in the presence and absence of Shield-1 (1 µM). The overlay image shows mDD (XFP, green), Mitotracker Orange (red), and Hoechst stain (blue). (B) Fluorescence micrographs of mDDc cells as in (A). (C) Flow cytometry of mDDn cells with Shield-1 (2 µM) or vehicle control after a 6 hour incubation. Cells were co-treated with cycloheximide (CHX, 5 µg/mL), MG132 (5 µM), or both CHX and MG132. (D) Flow cytometry of mDDn cells exposed to small molecules as in (C). Micrograph scale bars indicate 10 microns. Error bars represent ± S.E.M. (n = 3).

Mitochondrial DD cell lines, mDDn and mDDc, had a high fluorescence background in the absence of Shield-1 (Figure 2A and B). Colocalization of DD fusion with a Mito-tracker orange stain indicated proper targeting in both mitochondrial DD cell lines, mDDn and mDDc, via the ALDH2 matrix targeting sequence. While mDDn fluorescence was solely targeted to mitochondria in the presence and absence of Shield-1 based on colocalization of the fluorescent protein with the Mitotracker dye, the addition of Shield-1 caused fluorescence signal localized to both the mitochondria and cytoplasm in mDDc cells (Figure 2B).

Flow cytometry indicated that Shield-1 does not significantly affect the levels of mDDn (Figure 2C) contrasting the Shield-1 dependent regulation in the cytoplasm and nucleus. We used CHX and MG132 to probe whether this observation was related to production or degradation. Neither CHX nor MG132 treatment significantly affected the levels of DD fusions in mDDn cells in the absence of Shield-1 (p = 0.22, p = 0.12 respectively, Figure 2C). In mDDc cells there was a 1.8-fold increase in fluorescence after Shield-1 treatment and small fluorescence changes after treatment with CHX and MG132, suggesting a cytoplasmic fusion pool we observed with fluorescence microscopy (Figure 2D).

### Endoplasmic reticulum destabilizing domains

**Figure 3:**
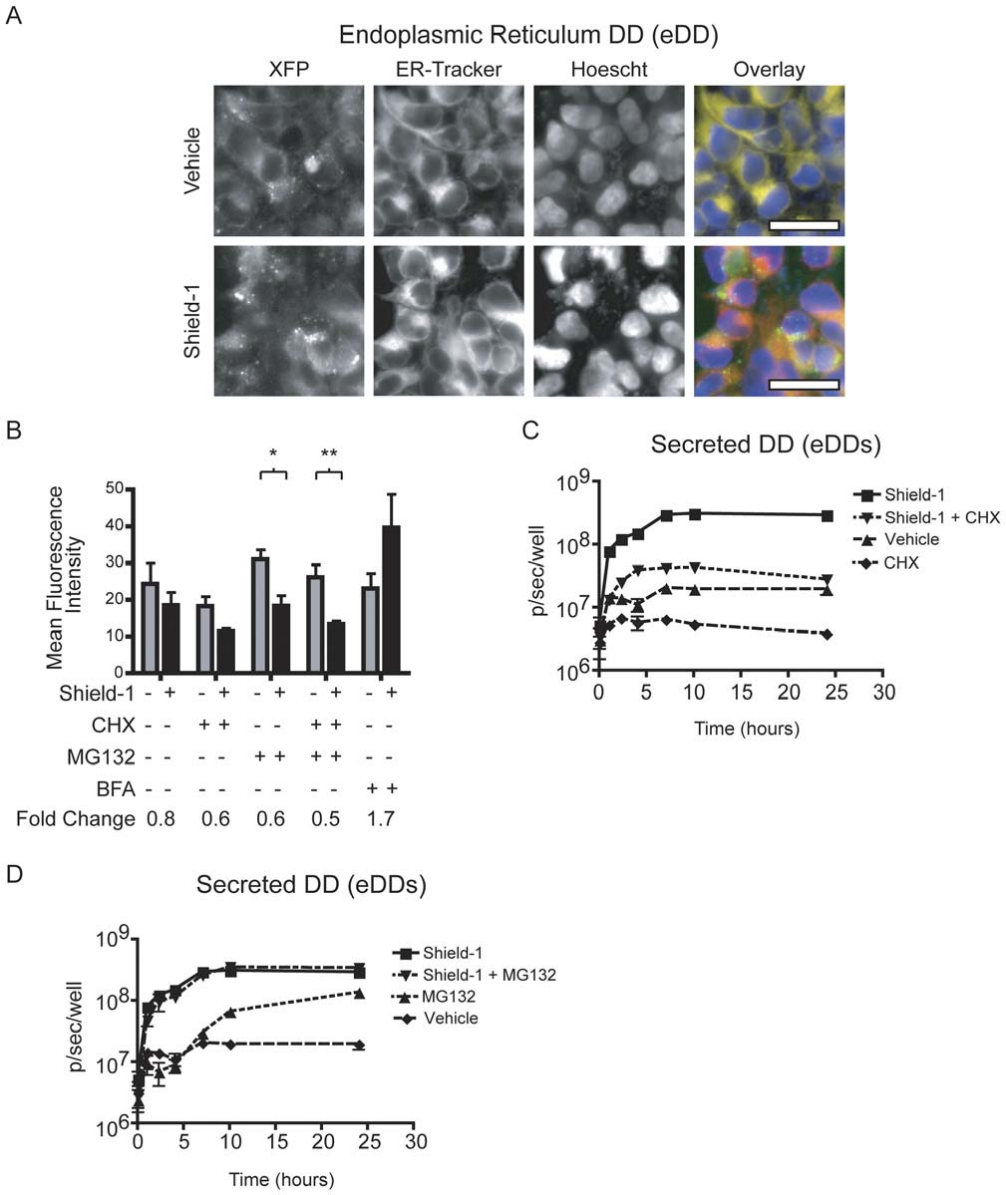
ER and secreted destabilizing domains. (A) Fluorescence micrographs of eDD cells. The overlay image shows eDD (green), ER-Tracker Red (red), and Hoechst stain (blue). (B) Flow cytometry of eDD cells with Shield-1 (2 µM) or vehicle control after a 6 hour incubation. Cells were co-treated with cycloheximide (CHX, 5 µg/mL), MG132 (5 µM), or brefeldin-A (BFA, 2.5 µg/mL). * P-value<0.05, ** P-value<0.005. (C) Bioluminescence quantification of media from eDDs cells after exposure to vehicle control, Shield-1 (1 µM), CHX (1 µg/mL), or co-treatment with both Shield-1 and CHX. (D) Bioluminescence quantification of media from eDDs cells after exposure to vehicle control, Shield-1 (1 µM), MG132 (1 µM), or co-treatment with both. Error bars represent ± S.E.M. (n = 3).

While Shield-1 largely did not affect DD levels in the mitochondria, we have previously demonstrated robust Shield-1 dependent regulation of secreted ER-targeted proteins such as GLuc, IL-2, and TNF-α [3]. As with the mitochondrial DDs, microscopy revealed the presence of fluorescence in both vehicle and Shield-1 treatment groups in eDD cells (Figure 3A). Clear colocalization of eDD fluorescence with an ER stain occurred in the absence of Shield-1 and small puncta were evident, suggesting protein aggregation. In the presence of Shield-1, colocalization with the ER was reduced (Figure 3A) and there was increased colocalization with the Golgi apparatus (Figure S3). Additionally, there was higher total intracellular eDD fluorescence in the absence rather than the presence of Shield-1 (Figure 3B). To address this observation we treated cells with brefeldin-A (BFA) to inhibit protein transport from the ER to the Golgi. BFA treatment caused intracellular fluorescence levels to rise 1.7-fold when treated with Shield-1 as analyzed by flow cytometry (Figure 3B). These data fit a model of Shield-1 induced stabilization and translocation of the eDD through the Golgi network and eventual secretion.

In the absence of Shield-1, treatment of eDD cells with CHX and MG132 did not cause a statistically significant reduction or increase in mean fluorescence intensity respectively (p = 0.18, p = 0.48, Figure 3B). This suggested that as in the mitochondria, there was little constitutive turnover of ER targeted DD fusions in the absence of Shield-1. However, treatment with Shield-1 or vehicle resulted in statistically significant differences in fold change (0.6, p<0.05) after co-treatment with MG132 and MG132/CHX (Figure 3B). This fold change with co-treatment of MG132 was likely a result higher initial fluorescence levels in the presence of MG132 prior to Shield-1 administration.

To support the above findings and since it is difficult to quantify fluorescent proteins extracellularly, we investigated the effects of Shield-1, CHX, and MG132 on the flux of an ER-targeted DD fused to a luminescent reporter protein. *Gaussia* luciferase is a secreted, ATP-independent luciferase that yields quantitative measures of protein levels in the extracellular space [21]. Intracellular and extracellular luciferase activity was monitored using bioluminescence after Shield-1 (1 µM) or vehicle treatment. As predicted by microscopy, intracellular levels of Gaussia luciferase were not greatly affected by Shield-1 while extracellular levels varied over 10-fold (Figure S4).

eDDs cells were treated at various time points with Shield-1 (1 µM) and/or a low dose of CHX (1 µg/mL). Co-treatment with CHX and Shield-1 attenuated extracellular levels of luciferase approximately 10-fold relative to Shield-1 treatment alone (Figure 3C). This indicated that it is primarily nascent proteins that are stabilized by Shield-1 and supported similar comparisons in cDD and nDD cells. Treatment with MG132 (1 µM) led to eventual extracellular accumulation of GLuc after 12 hours (Figure 3D), fitting the model that degradation inhibitors such as MG132 can facilitate correct folding and localization of misfolded substrates [22], [23], [24].

**Figure 4:**
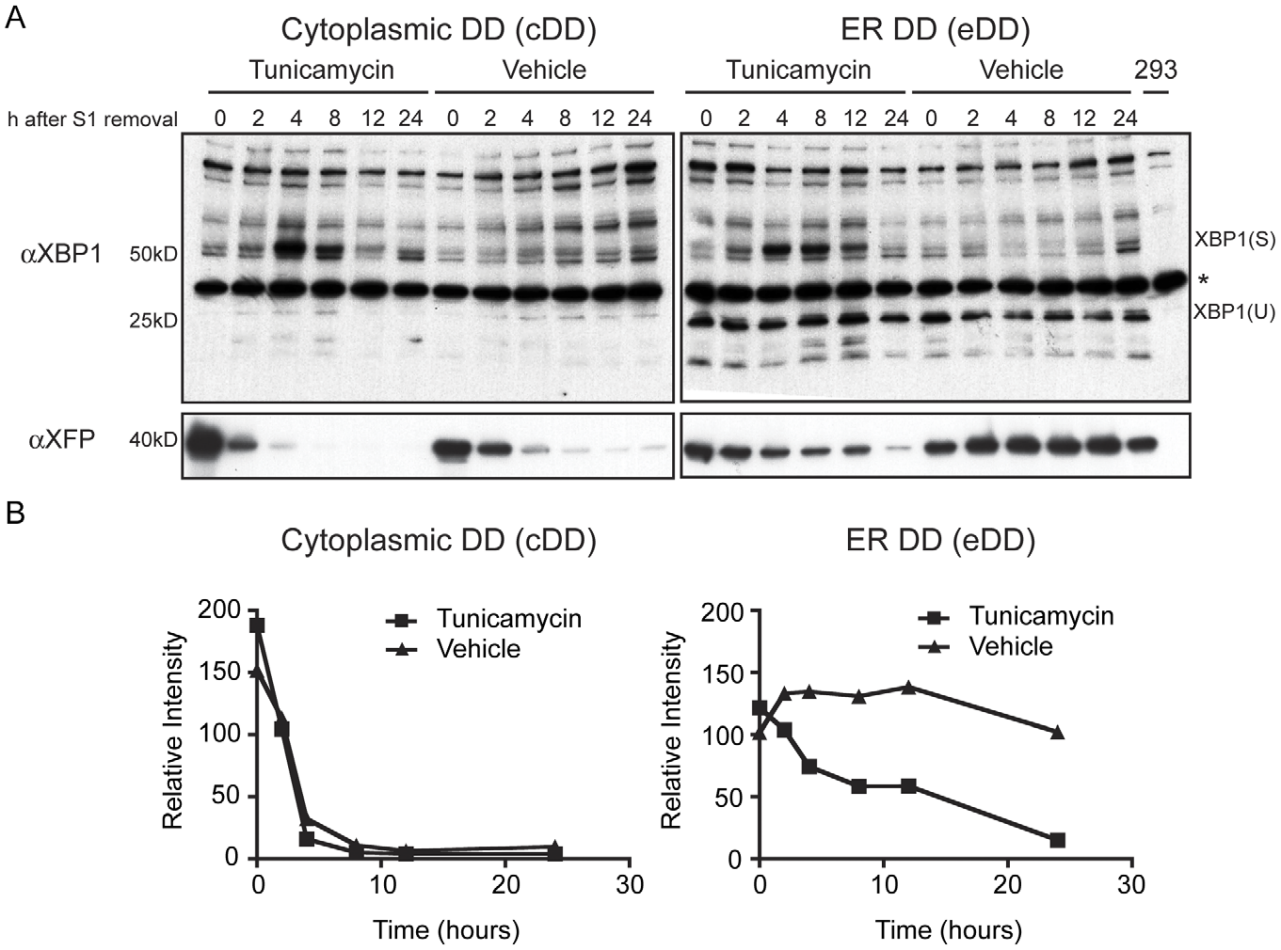
Immunoblot for XBP1 splicing and DD fusion levels after UPR induction with tunicamycin. cDD and eDD cells were cultured with Shield-1 for 96 hours prior to Shield-1 removal. Shield-1 washout with recombinant FBKP (5 µM) media occurred at various times in the presence or absence of tunicamycin (5 µM). (A) Blotting for XBP1 shows the spliced variant XBP(S) occurring at 4 and 8 hours when treated with tunicamycin. The blot was re-probed for XFP to assess the Shield-1 dependence of cDD and eDD. (*) A non-specific band in XBP1 staining is present in all lanes, including untransduced HEK293 cells (293). (B) Densitometry of DD fusion levels in the cytoplasm and ER after Shield-1 washout.

Our data suggested that compartment-specific folding and QC machinery were important to the functionality and degradation of the DD. Thus, we tested whether the folding environment in the ER could affect the intracellular levels of the eDD and vice versa (*i.e.* that the DD could affect the folding environment by stimulating a stress response). Specifically we were interested in whether the ER UPR could be induced by the removal of Shield-1, which has the effect of switching a cell from secreting to degrading the DD. When high levels of unfolded proteins are detected in the ER, mammalian cells can activate the UPR through three response pathways mediated by the proteins, IRE1αβ, PERK, and ATF6αβ/CREB-H [16]. IRE1 splicing of XBP-1 mRNA provides a time-dependent readout of ER stress [25]. The protein product of spliced XBP-1 mRNA, XBP(S), rises 4–8 hours after the addition of a stress agent such as tunicamycin, thapsigargan, or DTT in HEK293 cells [26]. We monitored the appearance of XBP(S) to determine whether the removal of Shield-1 would activate the ER UPR in eDD and control cDD cells. Cells were incubated first with Shield-1 for 96 hours to equilibrate the cells to the folded and secreted DD state followed by a timecourse of Shield-1 washout. No induction of the ∼50 kDa protein XBP(S) is seen 4 hours after Shield-1 washout in the either cDD or eDD cells (Figure 4A, vehicle). Addition of the ER stress agent tunicamycin induced a robust splicing response at 4 and 8 hours. These data suggest that the removal of Shield-1 was not a large enough insult to trigger the UPR as monitored by XBP-1 splicing.

The reverse question, whether the ER folding environment affects the levels of the eDD, was probed in the same experiment. Inducing the UPR with tunicamycin reduced intracellular levels of DD fusions in the ER as monitored using an αXFP immunoblot suggesting that UPR related increases in ER quality control machinery had significant effects on the levels of the mis/unfolded DD substrates present in the ER (Figure 4A & B). As expected cDD levels are highly sensitive to the washout of Shield-1 indicated by the deceasing amounts of DD-XFP present over time. eDD levels showed little Shield-1 sensitivity, supporting our earlier microscopy and flow cytometry data (Figure 3A & B). Taken together these data suggested that the ER harbored significant levels of mis/unfolded protein in the absence of Shield-1 and that the DD was sensitive to up regulation of the ER UPR.

## Discussion

Destabilizing domains have been fused to cytoplasmic, nuclear, and secreted proteins in many experimental systems, however their characteristics in the endoplasmic reticulum and mitochondria were previously unknown [3], [4], [5], [6], [7], [8], [9], [10]. As the mechanism of regulation is intrinsically related to the access of protein folding and degradation machinery, we reasoned that the cytoplasmically derived FKBP DD might exhibit variable levels based on subcellular localization. In this report we provide a baseline for future studies using this DD in subcellular compartments, show that the local protein quality control affects DD levels and show that the DD does not induce an IRE1 mediated stress response in the ER.

The DD functions in a chemically dependent manner in the cytoplasm, nucleus and through the secretory pathway. In these contexts small molecule inhibitors of translation, degradation, and secretion act on DD levels predictably, illustrating several dynamics of posttranslational regulation. Inhibiting translation with cycloheximide decreases the Shield-1 dependent dynamic range and blocking degradation with MG132 increases the basal fluorescence levels in the cells. After treatment of both Shield-1 and MG132, fluorescence levels are lower than in cells treated with Shield-1 alone, suggesting a decreased rate of protein translation and/or upregulated protein quality control machinery after MG132 treatment [27]. The mitochondria and ER, however, appear to be tolerant of elevated levels of the DD even in the absence of Shield-1.

The colocalization of fusion protein fluorescence with a mitochondrial stain shows proper targeting of both mDDn and mDDc. The pool of DD that is colocalized with mitochondria appears to be Shield-1 insensitive. We speculate that this may stem from the lack of protein QC machinery in the mitochondria that can recognize and degrade the DD in the absence of Shield-1, however, further biochemical studies such as gradient centrifugation will be necessary to prove that the mDD is fully translocated and intact in the mitochondrial matrix. After culture of mDDc cells for several weeks, inclusion bodies develop in a small population of cells suggesting cellular stress that we have not observed in any other DD-containing cell lines (Figure S5). Since protein homeostasis in the mitochondria is a balance between non-selective degradation by processes such as autophagy and selective degradation by peptidases and ATP-dependent proteases, the development of an orthogonal mitochondria-specific DD may be challenging, but also highly valuable given the importance of mitochondrial proteins in pathologic process such as aging and neurodegenerative diseases [28]. A less obvious application of a mitochondria-specific DD would be to function as a biosensor for compartmental protein QC activity as cells age, face pathogens or are subjected to other stresses.

High fluorescence levels of both mitochondrial DD cell lines in the absence of Shield-1 suggests that cytoplasmic QC machinery cannot degrade the DD fusions fully before mitochondrial localization. Whether proteins are cotranslationally inserted into the mitochondria or nascent polypeptides are released from ribosomes in the cytosol for posttranslational import (or are imported via a combination of both) remains an open question [29]. If the mDD was exposed to cytoplasmic degradation machinery before mitochondrial import, we might expect to see little signal in the absence of Shield-1 depending on the relative rates of synthesis, degradation, and import. Thus both cotranslational insertion into the mitochondria or chaperone-protected transport to the mitochondrial outer membrane channels are possible explanations for the accumulation of mDDn and mDDc in the mitochondria.

Microscopy of mDDc cells, in contrast with mDDn cells, indicates the presence of fluorescent proteins in both the cytoplasm and mitochondria when treated with Shield-1. Cytoplasmic localization of the mDDc fusions could be experimentally supported by an immunoblot that shows the ratio of cleaved fusions (mitochondrial) to uncleaved fusions (cytoplasmic). Thus, in the presence of Shield-1, uncleaved levels alone would rise. One potential reason for this dual localization is that the placement the rapidly folding Venus fluorescent protein N-terminally with respect to the DD reduces the efficiency of mitochondrial import, creating a cytoplasmic pool. In the absence of Shield-1 the defective importation is not observed because the cytoplasmic population of mDDc could be degraded. Co-treatment with MG132 in the absence of Shield-1 increases fluorescence levels of mDDc, suggesting that cytoplasmic proteasomal degradation of the protein is occurring. A second explanation is that Shield-1 binding of the DD when located on the C-terminus of the fusion protein causes a percentage of the proteins to be “unfolding incompetent,” and thus, import incompetent. In this case, Shield-1 would stabilize the protein such that the mitochondrial importation machinery cannot unfold the protein. Matoushek and co-workers have observed a similar phenomenon in yeast mitochondrial suspensions where treatment with a stabilizing ligand, methotrexate, can cause defective mitochondrial import of dihydrofolate reductase [30].

Our observations of the eDD provide valuable insights for future use of destabilizing domains in the ER. Immunoblot and microscopy show that a reservoir of eDD exists in the absence of Shield-1 at high intracellular levels that are comparable to protein levels in Shield-1 stabilized cDD cells. The addition of Shield-1 allows the secretion of DD fusions through the canonical secretion pathway as evidenced by treatment with brefeldin-A and CHX treatment significantly reduces luminescent protein secretion. Destabilizing domains in the ER may aggregate as suggested by puncta formation (Figure 3A) in a similar manner to another FKBP mutant that was used for conditional ER aggregation [31]. Though the DD does not have a large dynamic range of regulation within the ER itself, additional insights into ER regulation may be gained by determining the relative “age” of the DD fusions trapped in the ER with a photoactivatable fluorescent protein or pulse-chase experiment [32].

Ongoing projects in our lab are investigating whether there are any cellular adaptations that occur when cells are expressing the DDs. Here we show that the removal of Shield-1 did not cause XBP-1 splicing in eDD cells suggesting that the IRE1αβ pathway of the UPR is not induced acutely when the cell is challenged with unstable ER localized protein. One intriguing difference between the cDD and eDD is the elevated expression of XBP1(U) (Figure 4A). XBP1(U) is a negative feedback regulator of XBP1(S) by complexing with XBP1(S) and shuttling it out of the nucleus for degradation via a nuclear export sequence and degradation motif [26]. Thus, eDD cells may have adapted to high levels of mis/unfolded protein in the ER during their generation, allowing eDD cells to tolerate and degrade accumulated mis/unfolded substrates within the bandwidth of the ER quality control machinery and without activating the UPR.

Treatment with tunicamycin reduced the levels of DD fusions in the ER (Figure 4A and B). This indicates that UPR related increases in degradation and/or decreases in translation have significant effects on the levels of mis/unfolded DD substrates present in the ER. Decreased translation may be mediated by another unfolded protein response pathway such as PERK-dependent translational attenuation, which would be consistent with decreased levels of eDD-GFP in CHX treated cells shown in Figure 3B [33]. Future experiments monitoring intracellular and extracellular luciferase activity or a pulse chase analysis after tunicamycin treatment may demonstrate the predominate mechanism leading to decreased DD fusion levels. Regardless of mechanism, the DD is quite sensitive to local, compartment-specific protein quality control and greatly affected by the ER unfolded protein response.

The destabilizing domain technology has proven utility in many different experimental settings to predictably and conditionally tune protein levels in cells. These results may guide the use of the destabilizing domains in new experimental systems and provide a comprehensive baseline of expected regulation in the cytoplasm, nucleus, extracellular space, ER, and mitochondria. We find that the local protein QC environment in the ER affects the basal levels of the DD in the absence of Shield-1. This information may direct the future development of new DD-ligand pairs that can orthogonally regulate proteins in different cellular compartments. In addition to providing the ability to perturb cellular processes and pathways through direct fusion to proteins of interest, the destabilizing domains may eventually be used to facilitate insights into the endogenous machinery of protein homeostasis and degradation.

## Materials and Methods

### Retroviral Gene Expression

Various fluorescent proteins, YFP, GFP, and Venus [20] and a secreted luminescent protein *Gaussia* Luciferase (GLuc, [34]) and subcellular targeting sequences, SV40 nuclear localization sequence (NLS, [35]), *Gaussia* ER localization sequence (LS, [19]), ALDH2 mitochondrial targeting sequence (MTS, [18]) were genetically fused to a destabilizing domain (Table 1). All DDs were the F36V L106P mutant of human FKBP12, except the mDDc cell line that contained the F36V, E31G, R71G, K105E variant, the most robust C-terminal DD. These fusion genes were cloned into pBMN retroviral expression vectors containing blasticidin or puromycin drug resistance genes. Amphotrophic phoenix cell lines were plated at 2×10^6^ cells in a 10-cm dish 12 hours before transfection with pBMN vectors. Cells were transfected with Lipofectamine 2000 in Opti-MEM at a 2∶5 ratio (µg DNA ∶ µL cationic lipid). HEK293 human embryonic kidney cells (ATCC) were plated at 1×10^6^ cells per plate in a 10-cm dish and incubated with complete media (10% FBS, 10 units Pen/Strep) containing retrovirus and polybrene (4 µg/mL, Sigma) at 37°C overnight. At this time the retroviral media was removed and the cells were incubated with complete media. Blasticidin (10 µg/mL, Invitrogen) or puromycin (2 µg/mL, Invitrogen) was added to the media 48 hrs after transduction. Drug selection continued for 10 days.

### Microscopy

Stably transduced HEK293 cells with localized DD-XFPs were incubated overnight on chamber slides (Lab-TekII) in complete media with Shield-1 (1 µM) or ethanol vehicle control. The next day the cells were incubated with ER-Tracker red, MitoTracker orange, or BODIPY TR Ceramide (red fluorescent Golgi label) following company instructions (Molecular Probes, Invitrogen). All cells were incubated with Hoechst stain (1 µg/mL) for 5 minutes, washed with PBS containing calcium and magnesium, and imaged on an epifluorescent Axioscop2 (Zeiss) microscope and photographed using a c-mount camera (QImaging).

### Flow Cytometry

Stably transduced HEK293 cells were incubated with Shield-1 (2 µM), cycloheximide (CHX, 5 µg/mL, Sigma), MG132 (5 µM, Calbiochem), Brefeldin A (2.5 µg/mL, Sigma), or ethanol vehicle control for 6 hours. Cells were then dissociated using trypsin/EDTA (0.05%, Gibco) and incubated on ice. Fluorescence data was recorded using a FACS Calibur Flow Cytometer (BD Biosciences).

### Statistical Analysis

P values were calculated using a paired two-tailed T test. P values<0.05 were considered significant.

### Luciferase Assays

Cells containing DD-regulated secreted luciferase, eDDs cells, were plated in triplicate in a 96-well plate. Cells were treated at various times with non-toxic doses of vehicle (ethanol control), Shield-1 (1 µM), CHX (1 µg/mL), MG132 (1 µM), or co-treated with Shield-1 and CHX or MG132. The media from the eDDs cells was transferred to a new 96-well plate, coelenterazine (100 ng/mL, Nanolight) was added, and the luminescence was quantified using an In Vivo Imaging System (IVIS, Caliper Life Sciences).

### Immunoblotting

cDD and eDD cell lines were cultured with Shield-1 (1 µM) for 96 hours before being split to a 24-well plate. Shield-1 media was replaced at 0, 2, 4, 8, 12, and 24 hours with recombinant FKBP-containing media (5 µM) prior to collecting cells for western blot. In a duplicate group of wells, the media was replaced with recombinant FKBP and co-treated with tunicamycin (5 µM, Sigma). A gradient (4–20%) SDS-PAGE gel (Biorad) was run and protein was transferred to PDVF membrane (Millipore). Membranes were blocked in 10% dry milk for 1 hour and exposed to rabbit polyclonal anti-XBP1 (1 µg/mL, Abcam) antibody overnight at 4°C. The membranes were then washed in TBST buffer and exposed to anti-rabbit HRP conjugated secondary antibody (0.2 µg/mL, Molecular Probes). Chemiluminescence was performed using Immobilon Western Kit (Millipore). The antibodies were dissociated from the membrane with Restore Western Blot Stripping Buffer (Thermo Scientific) for 15 minutes and exposed to anti-XFP antibody (0.2 µg/mL, Clontech) following a similar procedure to the above. Densitometry of DD-XFP fusion levels were assessed using ImageJ software (NIH).

## Supporting Information

Figure S1 **Flow cytometry of DD fusion cell lines.** Each DD-containing cell line and a mitochondria-targeted Venus fluorescent protein cell line (no DD) was exposed to Shield-1 (2 µM) and assessed by flow cytometry for viral transduction efficiency post-antibiotic selection. Transduction efficiency was measured by the percentage of cells that were more fluorescent (FL-1) than 98% of untransduced HEK293 (the black bar represents that gated population).
(DOCX)

Figure S2 **Control cell lines treated with Shield-1 and various small molecule inhibitors.** (A) Flow cytometry of untransduced HEK293 cells with Shield-1 (2 µM) or vehicle control after a 6 hour incubation. Cells were co-treated with cycloheximide (CHX, 5 mg/mL), MG132 (5 µM), or both CHX and MG132 or brefeldin-A (BFA, 2.5 mg/mL). (B) MTS-Venus cells contain a mitochondria targeted XFP that is DD-free. Experimental conditions for flow cytometry are identical to those reported in (A). (C) Fluorescence micrographs of MTS-Venus cells. The overlay image shows (XFP, green), Mitotracker Orange (red), and Hoechst stain (blue). Micrograph scale bar indicates 10 microns. Error bars represent ± S.E.M. (n = 3).
(DOCX)

Figure S3 **eDD colocalizes with the Golgi apparatus in the presence of Shield-1.** Fluorescence micrographs of eDD cells. The overlay image shows eDD (green) and a ceramide Golgi Tracker (red). White arrows indicate colocalization of eDD with Golgi bodies after Shield-1 treatment.
(DOCX)

Figure S4 Intracellular and extracellular Gaussia luciferase bioluminescence from eDDs cells over time. Bioluminescence quantification of media (serum) or of washed eDDs cells after exposure to vehicle control or Shield-1 (S1, 1 µM).
(DOCX)

Figure S5 **Inclusion bodies in mDDc cells.** Fluorescence and bright field micrographs of mDDc cells after Sheild-1 and vehicle treatment. White arrows indicate inclusion bodies.
(DOCX)
