# Supplementary material for: Intracellular Context Affects Levels of a Chemically Dependent Destabilizing Domain
Source: PLoS One. 2012 Sep 12;7(9):e43297. doi: 10.1371/journal.pone.0043297 (PMC3440426; doi:10.1371/journal.pone.0043297)
Supplement: Figure S1 — Flow cytometry of DD fusion cell lines. Each DD-containing cell line and a mitochondria-targeted Venus fluorescent protein cell line (no DD) was exposed to Shield-1 (2 µM) and assessed by flow cytometry for viral transduction efficiency post-antibiotic selection. Transduction efficiency was measured by the percentage of cells that were more fluorescent (FL-1) than 98% of untransduced HEK293 (the black bar represents that gated population). (DOCX) [file pone.0043297.s001.docx]

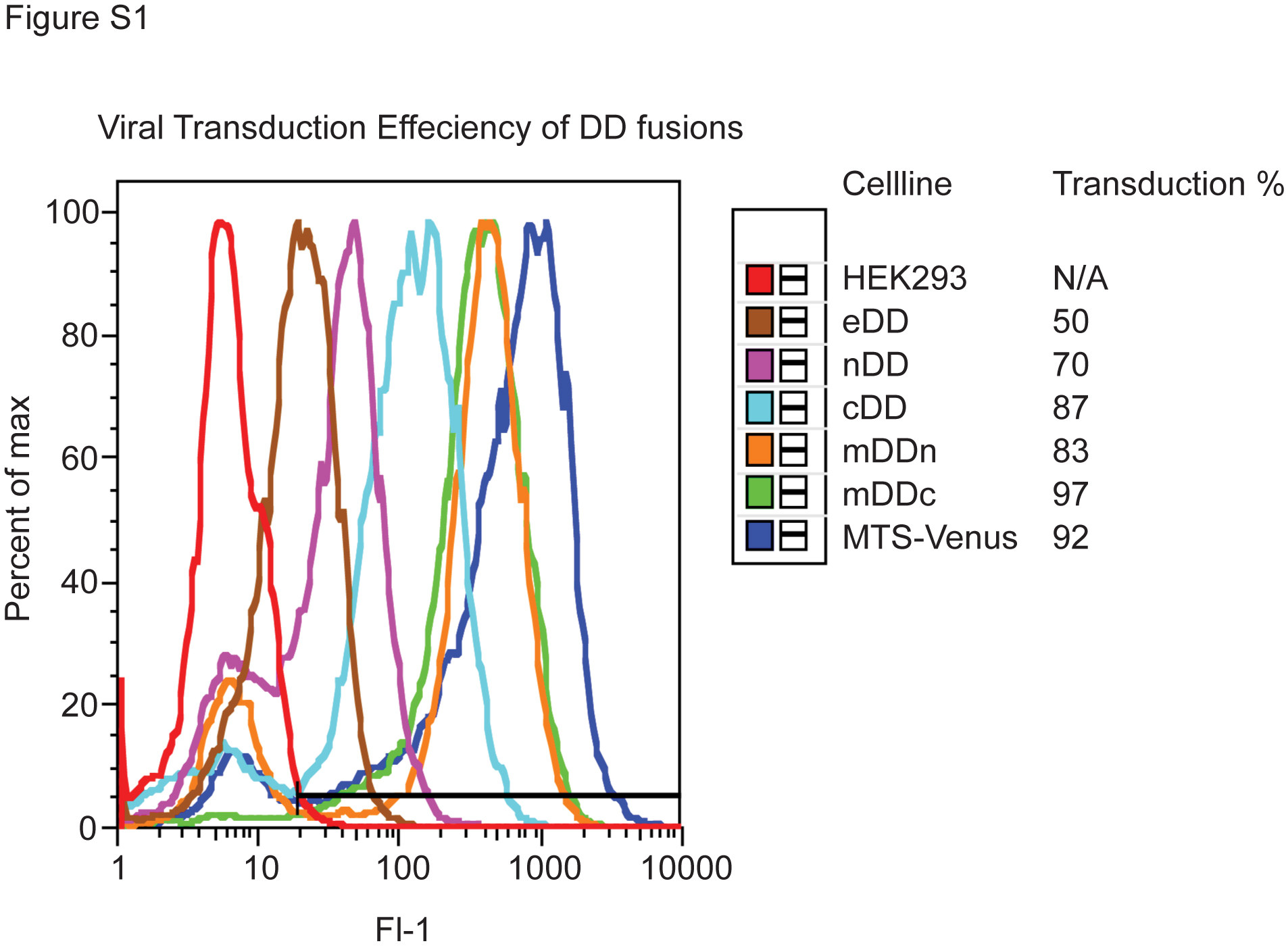


**Figure S1**. **Flow cytometry of DD fusion cell lines.** Each DD-containing cell line and a mitochondria-targeted Venus fluorescent protein cell line (no DD) was exposed to Shield-1 (2 μM) and assessed by flow cytometry for viral transduction efficiency post-antibiotic selection. Transduction efficiency was measured by the percentage of cells that were more fluorescent (FL-1) than 98% of untransduced HEK293 (the black bar represents that gated population).
