# Supplementary material for: Intracellular Context Affects Levels of a Chemically Dependent Destabilizing Domain
Source: PLoS One. 2012 Sep 12;7(9):e43297. doi: 10.1371/journal.pone.0043297 (PMC3440426; doi:10.1371/journal.pone.0043297)
Supplement: Figure S2 — Control cell lines treated with Shield-1 and various small molecule inhibitors. (A) Flow cytometry of untransduced HEK293 cells with Shield-1 (2 µM) or vehicle control after a 6 hour incubation. Cells were co-treated with cycloheximide (CHX, 5 mg/mL), MG132 (5 µM), or both CHX and MG132 or brefeldin-A (BFA, 2.5 mg/mL). (B) MTS-Venus cells contain a mitochondria targeted XFP that is DD-free. Experimental conditions for flow cytometry are identical to those reported in (A). (C) Fluorescence micrographs of MTS-Venus cells. The overlay image shows (XFP, green), Mitotracker Orange (red), and Hoechst stain (blue). Micrograph scale bar indicates 10 microns. Error bars represent ± S.E.M. (n = 3). (DOCX) [file pone.0043297.s002.docx]

**
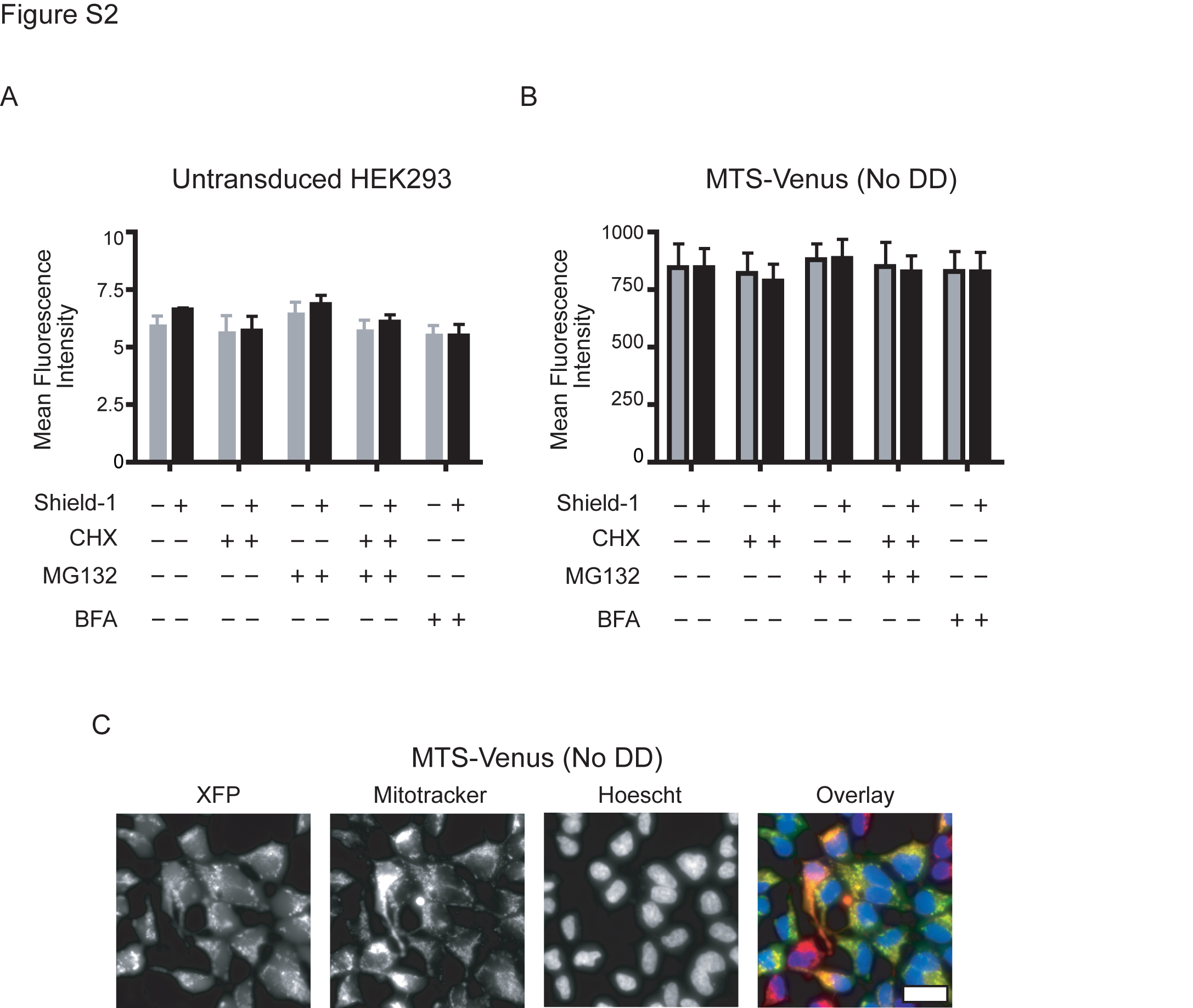
**

**Figure S2**. **Control cell lines treated with Shield-1 and various small molecule inhibitors.** (A) Flow cytometry of untransduced HEK293 cells with Shield-1 (2 μM) or vehicle control after a 6 hour incubation. Cells were co-treated with cycloheximide (CHX, 5 mg/mL), MG132 (5 μM), or both CHX and MG132 or brefeldin-A (BFA, 2.5 mg/mL). (B) MTS-Venus cells contain a mitochondria targeted XFP that is DD-free. Experimental conditions for flow cytometry were identical to those reported in panel (A). (C) Fluorescence micrographs of MTS-Venus cells. The overlay image shows (XFP, green), Mitotracker Orange (red), and Hoechst stain (blue). Micrograph scale bar indicates 10 microns. Error bars represent ± S.E.M. (n=3).
