# Supplementary material for: Intracellular Context Affects Levels of a Chemically Dependent Destabilizing Domain
Source: PLoS One. 2012 Sep 12;7(9):e43297. doi: 10.1371/journal.pone.0043297 (PMC3440426; doi:10.1371/journal.pone.0043297)
Supplement: Figure S3 — eDD colocalizes with the Golgi apparatus in the presence of Shield-1. Fluorescence micrographs of eDD cells. The overlay image shows eDD (green) and a ceramide Golgi Tracker (red). White arrows indicate colocalization of eDD with Golgi bodies after Shield-1 treatment. (DOCX) [file pone.0043297.s003.docx]

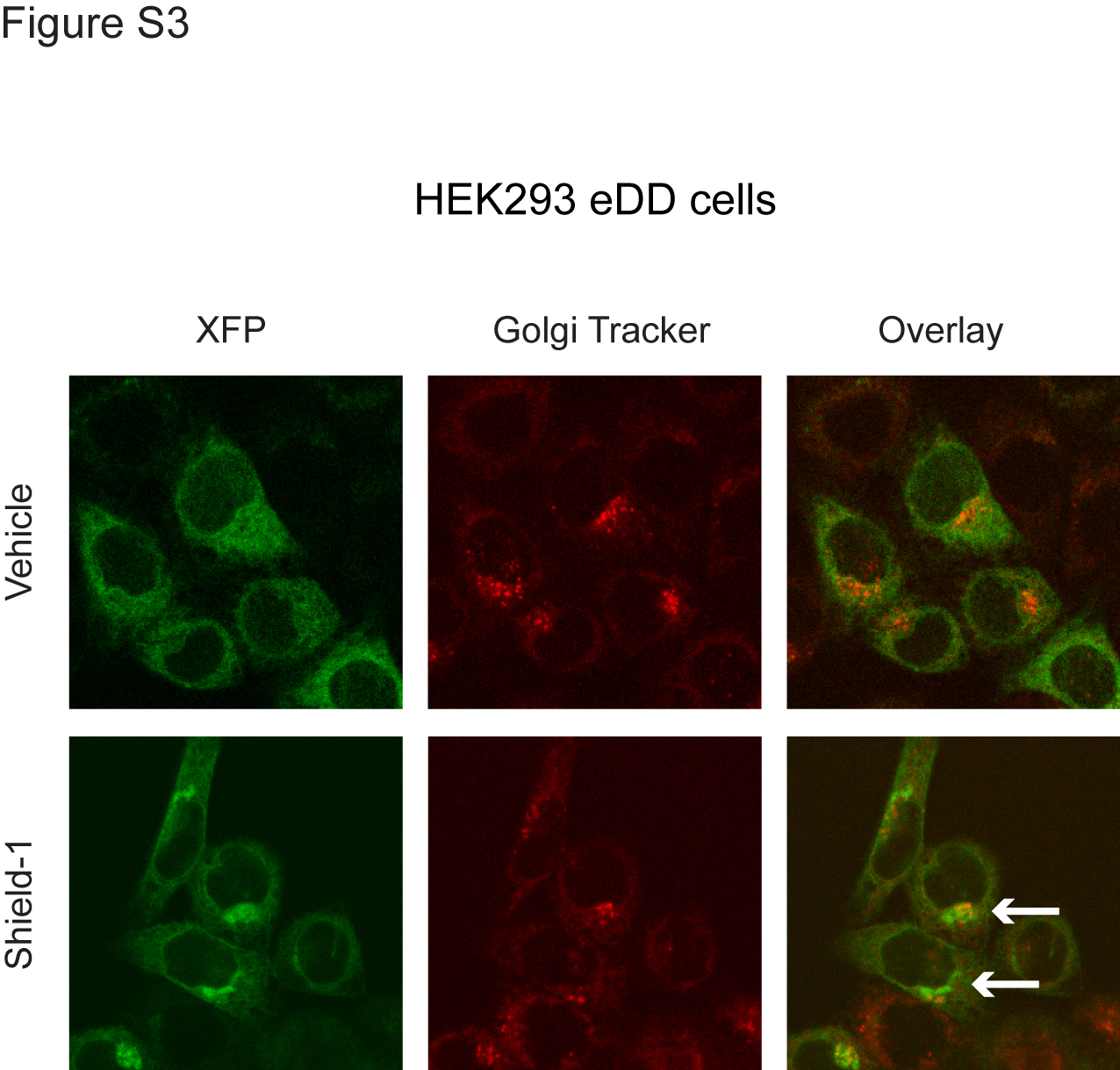


**Figure S3. eDD colocalizes with the Golgi apparatus in the presence of Shield-1.** Fluorescence micrographs of eDD cells. The overlay image shows eDD (green) and a ceramide Golgi Tracker (red). White arrows indicate colocalization of eDD with Golgi bodies after Shield-1 treatment.
