# Supplementary material for: Intracellular Context Affects Levels of a Chemically Dependent Destabilizing Domain
Source: PLoS One. 2012 Sep 12;7(9):e43297. doi: 10.1371/journal.pone.0043297 (PMC3440426; doi:10.1371/journal.pone.0043297)
Supplement: Figure S4 — Intracellular and extracellular Gaussia luciferase bioluminescence from eDDs cells over time. Bioluminescence quantification of media (serum) or of washed eDDs cells after exposure to vehicle control or Shield-1 (S1, 1 µM). (DOCX) [file pone.0043297.s004.docx]

Figure S4

**Figure S4.**  Intracellular and extracellular Gaussia luciferase bioluminescence from eDDs cells over time. Bioluminescence quantification of media (serum) or of washed eDDs cells after exposure to vehicle control or Shield-1 (S1, 1 μM).
