# Supplementary material for: Intracellular Context Affects Levels of a Chemically Dependent Destabilizing Domain
Source: PLoS One. 2012 Sep 12;7(9):e43297. doi: 10.1371/journal.pone.0043297 (PMC3440426; doi:10.1371/journal.pone.0043297)
Supplement: Figure S5 — Inclusion bodies in mDDc cells. Fluorescence and bright field micrographs of mDDc cells after Sheild-1 and vehicle treatment. White arrows indicate inclusion bodies. (DOCX) [file pone.0043297.s005.docx]

Figure S5


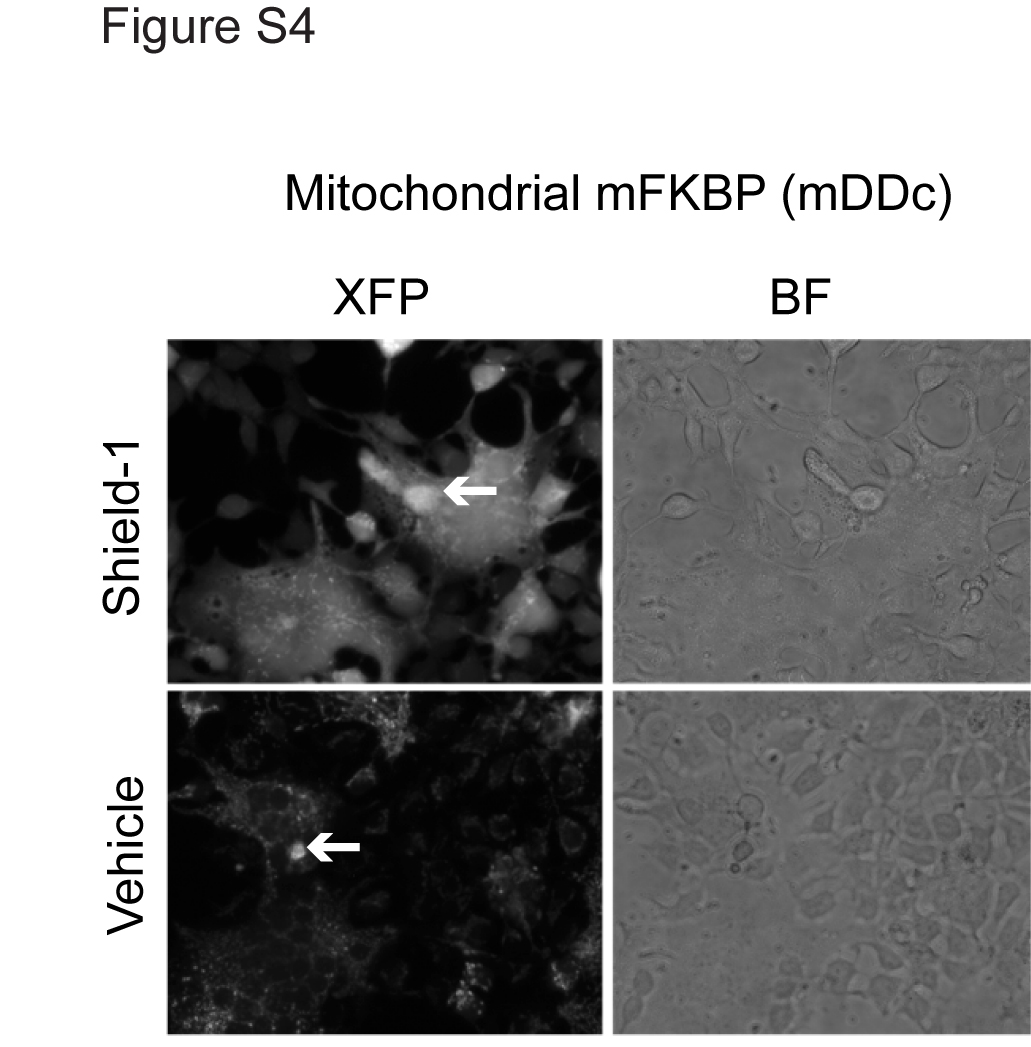


**Figure S5. Inclusion bodies in mDDc cells.**  Fluorescence and bright field micrographs of mDDc cells after Sheild-1 and vehicle treatment. White arrows indicate inclusion bodies.
